# Research progress of macrophage ferroptosis in inflammatory bowel disease and inflammation-cancer transformation

**DOI:** 10.3389/fimmu.2025.1658280

**Published:** 2025-09-23

**Authors:** Siyu Chen, Jing Ma, Junling Tang, Yang Yang, Shiwen Zhou, Peimin Feng

**Affiliations:** ^1^ School of Clinical Medicine, Chengdu University of Traditional Chinese Medicine, Chengdu, China; ^2^ Hospital of Chengdu University of Traditional Chinese Medicine, Chengdu, China

**Keywords:** ferroptosis, macrophages, inflammatory bowel disease, colitis-associated colorectal cancer, iron homeostasis

## Abstract

The pathophysiology of inflammatory bowel disease (IBD), a chronic intestinal inflammatory disease, is tightly associated with immunological dysregulation, intestinal flora abnormalities, and intestinal epithelial cell destruction. Ferroptosis—a non-apoptotic cell death form that differs from the standard apoptotic mode—plays a significant regulatory role in the development of IBD through iron-dependent lipid peroxide accumulation. Iron serves as a critical component for maintaining the normal function of macrophages. Macrophages have been demonstrated to play multifaceted roles in the pathogenesis and progression of inflammatory bowel disease. The iron metabolism within macrophages may potentially influence the development of IBD and colitis-associated cancer. This paper summarizes the present research on ferroptosis and macrophages and their related molecular mechanisms. It also discusses the interactive function of macrophage ferroptosis in the development of IBD and inflammatory-cancer transformation. The development of new theoretical foundations and intervention techniques for the prevention and treatment of IBD and colitis-associated colorectal cancer will be facilitated by the growth of this research area.

## 1 Introduction

Inflammatory bowel disease (IBD) is one of the chronic, progressive, and recurrent intestinal diseases, including ulcerative colitis (UC) and Crohn’s disease (CD). Even though the pathogenesis’s causes and processes are yet unknown, they are intimately linked to immunological dysfunction, intestinal mucosal barrier impairment, and inflammatory damage (1). The incidence of IBD is still increasing every year, and some studies have shown that some patients with IBD have the possibility of further transformation to colon cancer (2). Therefore, it is essential to study the pathogenesis behind it to prevent and treat this disease. Intestinal epithelial cells (IECs), which have a very fast rate of cell renewal and are typically characterized by significant epithelial erosions, have been shown to play a key role in IBD in recent studies. This phenomenon is common in a variety of intestinal disorders, such as IBD. Natural apoptosis of IECs is essential to maintaining their functionality, which helps to maintain their ability to renew continuously and the balance of tissue homeostasis. However, when the IECs undergo excessive apoptosis, it exacerbates the elevated intestinal permeability and the dysfunction of the intestinal mucosal barrier, a process that is considered to be a central factor in the development of IBD (3).

Macrophages, which are abundant in the digestive tract, are crucial for preserving immunological and inflammatory homeostasis in IBD (4). Macrophages can affect IBD through various metabolic pathways such as glucose metabolism, fatty acid metabolism, amino acid metabolism, etc. (5–8). Macrophages in IBD also display metabolic traits that set them apart from other forms of inflammatory diseases. Intestinal macrophages promote IECs by functioning as metabolic symbionts in addition to immune protection. This occurs by stimulating cells to promote intestinal epithelial differentiation and homeostasis, thereby synergizing immunomodulatory and tissue homeostatic maintenance functions (9), and their persistent absence leads to intestinal vascular-neurological abnormalities, impaired barrier function, and intestinal motility dysfunction (10). Scott and his team showed that (11) antibiotic-induced microbiota disruption promotes the activation of glycolysis and the oxidative phosphorylation (OXPHOS) pathway in colonic macrophages. At the same time, the gastrointestinal tract differs in various types of microenvironments, and these differences play a key role in delineating the metabolic specificity of intestinal macrophages. Macrophages in the gut are usually polarized into two phenotypes, M1 and M2, and under certain conditions, the two phenotypes can also transform into each other. M1 macrophages promote inflammation by recruiting leukocytes, activating the vascular endothelium and the immune system, whereas M2 macrophages inhibit inflammation by scavenging dead cells, producing anti-inflammatory factors, and inhibiting leukocyte recruitment (12–14). Thus, macrophages control the progression of IBD by promoting and inhibiting the inflammatory response.

Targeting the intestinal system, IECs death weakens the structural integrity of the gut, leading to damage of the physical barrier by oxidative stress. As interactions between cells and their environment continue, other barriers in the gut are successively compromised, ultimately triggering a series of abnormalities in gut function (15). In addition, programmed cell death patterns in the gut have a significant impact on tissue repair processes, an effect that predisposes to an increased long-term risk of inflammation transitioning to intestinal fibrosis and cancer (3). Therefore, it is particularly important to illustrate the mechanism of ferroptosis and its role in IECs, and the imbalance of iron homeostasis in macrophages can also influence the progression of IBD to a certain extent. Dysregulation of iron metabolism can affect macrophage cytokine release and macrophage polarization, thereby influencing the immune system and inflammation (16). In recent years, the interaction between ferroptosis and macrophages has attracted much attention. Ferroptosis and macrophages are jointly involved in the pathogenesis of IBD. Therefore, starting from the regulation of macrophages and their polarization and the inhibition of ferroptosis has an important role in the treatment of IBD (17, 18) and is a potential way to control inflammation, immune response, and influence the progression of IBD. In this review, we illustrate the pathological mechanisms of ferroptosis as well as discuss the potential role of iron homeostasis in macrophages in the treatment of IBD.

## 2 Ferroptosis

### 2.1 Overview of ferroptosis

**Figure 1:**
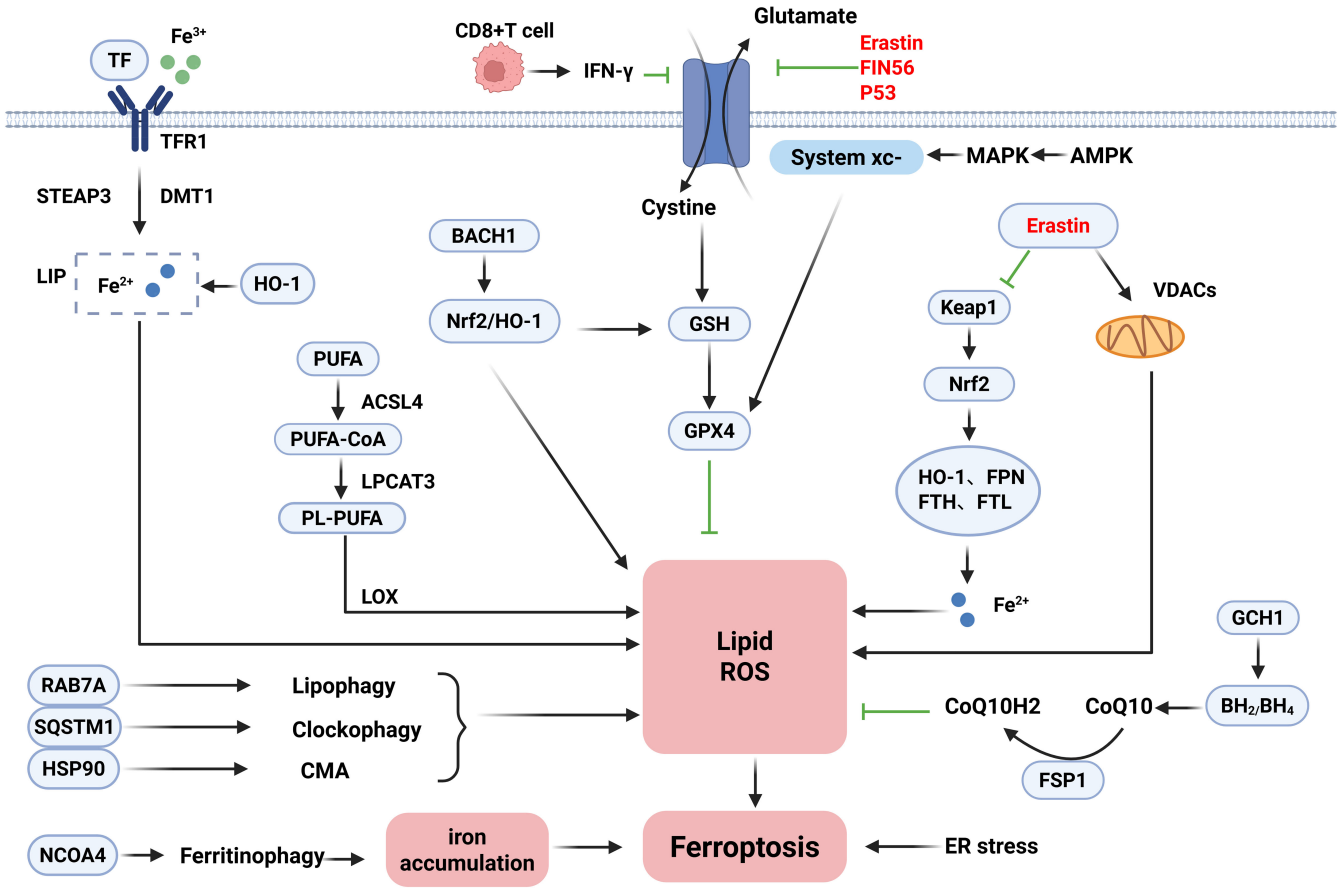
Mechanisms of ferroptosis.

Ferroptosis is a form of iron-dependent non-apoptotic cell death first proposed by Dixon (19) et al. in 2012, which is distinguished from traditional cell death modalities such as apoptosis, necrosis, and autophagy and is usually characterized by differences in biochemical, morphological, and genetic aspects. Ferroptosis is mostly characterized biochemically by iron accumulation and lipid peroxidation, and the accumulation of excess iron generates large amounts of reactive oxygen species (ROS) via the Fenton reaction, leading to redox damage and thus promoting cellular ferroptosis (19, 20). Iron metabolism-related genes such as transferrin (TF) and ferrous transfer protein (FPN) affect iron uptake in erythroid cells by regulating iron homeostasis.TF binds to the transferrin receptor (TFR) on the cell surface to form a complex, which is mediated by receptor-mediated internalization into the endosomes and then reduces Fe^3+^ to Fe^2+^ in an acidic environment via the six-transmembrane epithelial antigen of prostate 3 (Steap3). Eventually, it is transported across the membrane by the divalent metal transporter 1 (DMT1) to the cytoplasmic iron pool (LIP) for storage (21, 22). Compared with the traditional death mode, ferroptosis is usually characterized by a necrotic morphology, which often shows abnormal mitochondrial morphology under the microscope, such as shrinkage of mitochondria, accompanied by an increase in membrane density and a decrease or even disappearance of cristae (19, 23, 24). Previously, however, it has been shown that compounds such as erastin can destroy tumor cells through non-apoptotic cell death mechanisms, and RSL3 has also been found to contribute to such cell death patterns (25). The function of erastin can be dependent on voltage-dependent anion channels (VDAC). It is worth noting that ligands for VDAC can selectively trigger non-apoptotic cell death processes against some tumor cells carrying activating mutations in the RAS-RAF-MEK pathway (24). Iron, as a trace element, is involved in the normal physiological functions of the human body. Both iron deficiency and iron overload can affect the health of the organism (26), so maintaining the balance of iron homeostasis in the body is of great value in promoting the healing of diseases. Therefore, it is particularly important to illustrate the role of ferroptosis mechanisms in IBD. The detailed mechanisms of ferroptosis are shown in **Figure 1**.

### 2.2 Mechanism of ferroptosis

Ferroptosis is a form of cell death regulated by multiple pathways and mechanisms in a coordinated manner. Its occurrence depends on complex processes such as iron metabolism disorders, lipid peroxidation, and the interaction of autophagy regulatory networks. Therefore, the following section describes the mechanisms of induction, inhibition, and bidirectional regulation of ferroptosis. The core of the ferroptosis induction mechanism lies in the disruption of intracellular iron homeostasis and the accumulation of lipid peroxidation. Under normal conditions, intracellular iron metabolism is a complex physiological process. Intracellular iron homeostasis is maintained in balance through the regulation of the iron transport system. In contrast, the ferroptosis process is precisely regulated by a variety of iron metabolism-related regulatory factors, and iron uptake, storage, exocytosis, and turnover and utilization affect the sensitivity of cells to ferroptosis. Therefore, the homeostasis of iron metabolism and the regulation of ferritin may become important regulatory mechanisms of ferroptosis (27, 28). Ferroptosis sensitivity is modulated by iron dysregulation through multiple pathways: Extracellular Fe^3+^ bound to TF enters cells via TFR1-mediated endocytosis. It is then reduced to Fe^2+^ by prostate six-transmembrane epithelial antigen 3 (STEAP3) and transported to the LIP by DMT1. In addition to TF-mediated iron uptake, non-transferrin-bound iron (NTBI) is transported by solute carrier family 39 member 14 (SLC39A14), which aids in the activation of ferroptosis (29–31). Significantly, ferritinophagy plays a key role in regulating intracellular iron levels. Nuclear receptor coactivator 4 (NCOA4) acts as a specific receptor that delivers ferritin to autophagosomes. Through multiple pathways, autophagy-related proteins including RAB7A, SQSTM1, and HSP90 further promote lipid peroxidation during ferroptosis (32–34). In addition, ferroptosis execution also depends on lipid metabolism and peroxidation. Lipid peroxidation is brought on by excess iron through ROS produced by the Fenton reaction. Polyunsaturated fatty acids (PUFAs), critical components of cell membranes, are particularly vulnerable to ROS attack due to their structural characteristics (35). The production of PUFA-containing phospholipids (PUFA-PLs) and the metabolism of PUFAs have a major impact on ferroptosis sensitivity. Key enzymes acyl-CoA synthetase long-chain family member 4 (ACSL4) and lysophosphatidylcholine acyltransferase 3 (LPCAT3) drive PUFA incorporation into phospholipids: ACSL4 converts PUFAs to PUFA-CoAs, whereas LPCAT3 catalyzes PUFA-PL formation, ultimately inducing ferroptosis (36, 37). Therefore, downregulating ACSL4 or knocking down LPCAT3 represents a potential therapeutic strategy. Additionally, mitochondrial dysfunction exacerbates ferroptosis by generating excessive ROS and lipid peroxides. Endoplasmic reticulum (ER) stress and mitochondrial regulators also participate in this process (38). In immune regulation, activated CD8^+^ T cells trigger tumor cell ferroptosis by secreting γ-Interferon (IFN-γ) to downregulate SLC3A2/SLC7A11 expression, thereby enhancing antitumor immunity (39).

The core of the ferroptosis defense mechanism lies in the multilevel regulation of the antioxidant defense system: The system xc−/GSH/GPX4 axis is the most prominent pathway to inhibit the ferroptosis system. System xc− is a heterodimer composed of SLC7A11 and SLC3A2 that imports cystine. This cystine is reduced to cysteine for glutathione (GSH) synthesis. GPX4 utilizes GSH to reduce lipid peroxides; loss of GPX4 activity directly triggers ferroptosis (40). Notably, GTP cyclohydrolase 1 (GCH1) is a recently identified ferroptosis suppressor independent of GPX4. GCH1 and its metabolite tetrahydrobiopterin (BH4) form the GCH1-BH4-DHFR pathway. This pathway exerts antioxidant effects by generating BH4 and its derivative BH2. Overexpressing GCH1 significantly reduces damage in GPX4-deficient cells (41). Apoptosis-inducing factor mitochondria-associated 2 (FSP1/AIFM2) protects against GPX4 deficiency-induced ferroptosis. FSP1 catalyzes NAD(P)H-dependent coenzyme Q10 (CoQ10) regeneration, establishing the FSP1-CoQ10-NAD(P)H pathway. This system cooperates with GPX4 and GSH to inhibit phospholipid peroxidation and ferroptosis (42, 43). Nuclear factor erythroid 2-related factor 2 (Nrf2) is a master transcription factor regulating antioxidant responses in iron and lipid metabolism. As the specific receptor for Kelch-like ECH-associated protein 1 (KEAP1), Nrf2 balances oxidative stress through the KEAP1-Nrf2-GPX4 axis (44). Adipose-derived stem cell (ADSC) exosomes also suppress inflammation, oxidative stress, and ferroptosis by upregulating Nrf2 and GPX4 (45). In addition, Chen et al. (46) pointed out that the activation of multiple pathways in the inflammatory signaling pathway, such as JAK-STAT, and NF-κB, influences iron metabolism and lipid peroxidation, closely linking to ferroptosis. It has been shown that some novel targeted drugs can effectively intervene in ferroptosis through the above inflammatory pathways.

In the process of investigating the mechanisms related to ferroptosis, in addition to the unidirectional induction and inhibition mechanisms, studies have illustrated the dynamic balancing roles of two key molecules in ferroptosis, P53 and HO-1. P53 is a tumor suppressor gene that mediates cell cycle inhibition, apoptosis, and senescence and participates in metabolic activities (47). On the one hand, P53 can affect GPX4 activity by downregulating cystine expression, leading to reduced cellular antioxidant capacity, ROS accumulation, and ferroptosis (48). On the other hand, P53 exhibits an inhibitory effect on ferroptosis in some cells by decreasing system xc− activity or regulating GSH metabolism via the P53–P21 axis. Taken together, the regulation of ferroptosis by P53 may be bidirectional (40). Significantly, HO-1 is closely associated with ferroptosis and oxidative stress and can act as a key mediator to induce ferroptosis by catalyzing iron accumulation and cellular redox mechanisms (49, 50). Furthermore, treatment with the ferroptosis inhibitor ferrostatin-1 reduces HO-1 levels, thereby alleviating iron overload. This occurs because decreased HO-1 lowers ferritin and TF levels regulated by it (51).

In summary, the regulatory network of ferroptosis is characterized by a high degree of complexity and dynamic equilibrium. The fine regulation between induction and inhibition mechanisms determines the fate of cells, and the bidirectional regulation of molecules such as P53 and HO-1 further increases the dimension of regulation. In-depth analysis will not only help to illustrate the biological nature of ferroptosis but also provide a theoretical basis and potential targets for the development of therapeutic strategies against inflammation, cancer, neurodegenerative diseases, and ischemia–reperfusion injury.

### 2.3 IBD and ferroptosis

There is increasing evidence that programmed cell death plays an important role in the development of intestinal diseases, causing damage to tissues such as the intestinal mucosa and thus exacerbating the inflammatory response, there may even be a long-term risk of transformation of inflammation to cancer. It has been shown that ferroptosis is involved in the death of IECs in IBD and that ferroptosis is involved in the inflammatory response in IBD through lipid peroxidation, iron deposition, and excessive ROS, ultimately leading to IECs death. Inflammatory response, which ultimately leads to IECs death and extensive epithelial erosion (3, 52, 53). There is a link between ROS production and IBD and its cancerous progression. In addition, iron may have a direct effect on IECs function or create a pathological environment in the gut that can induce stress-related apoptosis in IECs by altering microbial homeostasis in the gut. Essential features of ferroptosis, including elevated levels of lipid peroxidation, GSH depletion, GPX4 inactivation, and disturbances in iron homeostasis, are observed in intestinal tissues from IBD patients as well as in animal models of IBD (54–56).

Ferroptosis-related molecules, genes, and proteins are closely associated with the development of IBD. Recently, Nrf2/HO-1 has been recognized as one of the potential targets for the treatment of IBD, which slows down IECs ferroptosis by reducing intestinal inflammation and injury by maintaining redox homeostasis (57). In addition, Nrf2, P53, and ATF3 can all affect GSH synthesis by mediating SLC7A11 expression (48, 58). BACH1 modulates inflammation and oxidative stress via the Nrf2/HO-1 pathway. Silencing this gene enhances HO-1 expression, thereby suppressing ROS generation (59). Reduced BACH1 protein levels are also associated with the upregulation of genes involved in the GSH synthesis pathway. Furthermore, this protein suppresses the transcription of ferritin and FPN genes, inducing cellular ferroptosis (59, 60). Targeted inhibition of ACSL4 treatment restores GPX4 expression, reduces COX2 expression, and decreases lipid peroxidation can effectively alleviate lipopolysaccharide (LPS)-induced ferroptosis and inflammation, thereby improving LPS-induced IECs dysfunction (61). It has been shown that NEDD4L deficiency promotes IECs ferroptosis by inhibiting GPX4 expression through decreasing SLC3A2 expression. The ferroptosis inhibitor reduces colitis susceptibility in NEDD4L-deficient mice, and thus it can be a therapeutic target for IBD by maintaining intestinal homeostasis (62). Furthermore, the phosphorylation level of STAT3 was downregulated in IEC-6 cells treated with H2O2, and Fer-1, a ferroptosis inhibitor, was able to restore and reactivate the phosphorylation status of STAT3. Moreover, H_2_O_2_ showed a cumulative effect on the degree of ferroptosis when combined with STAT3 phosphorylation inhibitors (54).

Interestingly, iron metabolism in IBD exhibits a paradoxical pathology: Approximately 60%-80% of patients develop iron deficiency due to chronic blood loss, reduced iron intake, and impaired intestinal absorption. Hepcidin inhibits intestinal iron absorption by degrading the iron exporter FPN on enterocytes. Meanwhile, intestinal macrophages accumulate intracellular iron from increased erythrophagocytosis of red blood cells, generating ROS through the Fenton reaction, activating inflammasomes and releasing pro-inflammatory factors, thus forming a vicious cycle of inflammation and iron overload (63, 64). Conventional iron supplementation corrects anemia but can also worsen intestinal inflammation. Oral iron supplements lead to the accumulation of free iron in the gut, promoting the proliferation of pathogenic microorganisms and intestinal flora imbalance, and simultaneously inducing mucosal lipid peroxidation damage. In contrast, iron chelators reduce local free iron concentrations within macrophages, and this shift promotes macrophage polarization from the pro-inflammatory M1 phenotype toward the anti-inflammatory M2 phenotype, thereby alleviating oxidative stress. However, it may exacerbate the state of systemic iron deficiency (64, 65). Diets rich in dietary lipids or high in dietary iron contribute to a decrease in GPX4 activity and thus increase the risk of IBD (66), whereas iron chelators and iron replacement therapies ameliorate IBD by decreasing lipid peroxidation and modulating gut flora (67, 68). In addition, the AHR repressor (AHRR) is a transcription factor that promotes intestinal immune response, and inhibition of AHRR expression can improve redox imbalance and lipid peroxidation in intestinal intraepithelial lymphocytes (IELs) to slow the progression of IBD (69). Exosomes secreted by human umbilical cord MSCs (hucMSC-Ex) possess the ability to inhibit ferroptosis by reducing the accumulation of lipid peroxidation products and enhancing the levels of GPX4 and GSH *in vivo*, a process that contributes to the reduction of intestinal inflammation and facilitates tissue damage repair (70). Most importantly, when iron homeostasis is imbalanced, excess iron is absorbed through the digestive tract and accumulates in the colon, leading to intestinal dysbiosis and probiotic attenuation, triggering ferroptosis mechanisms and inducing inflammatory responses (68). Animal studies have demonstrated that dysbiosis is a key pathology in iron overload-promoted IBD: Colony clearance exacerbates ferroptosis-associated inflammation, whereas colony remodeling reverses this pathology (71, 72). Moreover, there is evidence that bile acid metabolites produced by intestinal flora can also downregulate ferroptosis proteins, reverse iron homeostatic imbalances, and repair the intestinal barrier to alleviate UC via the Nrf2/GPX4 pathway (73, 74). NOX1, which is highly expressed in colon tissues, can mediate IBD and carcinogenesis through a dual mechanism of action (75, 76), so by regulating NOX1, this substance promotes ferroptosis and cancer cell death. In conclusion, the impact of iron metabolism on the intestinal tract is contradictory. Therefore, it is crucial to regulate iron homeostasis in clinical practice to maintain iron metabolism. Maintaining iron homeostasis provides an important theoretical basis and direction for the development of new therapies targeting ferroptosis in IBD in future clinical practice.

## 3 Macrophages and ferroptosis

### 3.1 Mutual regulation of macrophage and ferroptosis

During the phase of inflammation resolution, macrophages phagocytose programmed apoptotic cells, a process called efferocytosis. Efferocytosis is one of the main roles in alleviating inflammation and restoring tissue homeostasis, and altered metabolic functions in macrophages can modulate efferocytosis to some extent (77). Macrophages are tasked with the removal of ferroptosis cells. The presence of ferroptosis cells can activate macrophage-related functions or contribute to inflammatory response generation and macrophage recruitment by activating inflammatory pathways (78). In addition, gut flora metabolites inhibit ferroptosis via receptor modulation of macrophages and affect the differentiation and function of cells associated with immune regulation and inflammatory response, respectively (79). FPN1 is one of the many iron-homeostatic proteins that influence the function of the immune system. FPN1-deficient macrophages cause a significant elevation of TNF-a and IL-6, and their inflammatory factor aberrations are closely associated with imbalances in cellular iron homeostasis (80). IFN-γ significantly enhanced the iron export effect in Salmonella-infected phagocytes by downregulating TFR1-mediated iron uptake and upregulating the expression of the iron-exporting protein FPN1. This cytokine synchronously reduces intracellular iron reserves, which both limits intracellular iron acquisition by pathogenic bacteria and promotes NO and TNF-α synthesis through the regulation of iron homeostasis, forming a multilayered immune response mechanism (81). Macrophage migration inhibitory factor (MIF) acts as an inflammatory cytokine. It regulates macrophage migration and mediates pathological processes in the tumor microenvironment. Exosomal MIF enhances macrophages’ resistance to ferroptosis by decreasing ROS levels in the cells. Simultaneously, it balances cell survival and intracellular oxidative damage (82). Nitric oxide synthase 2 (NOS2) also plays a critical role in host defense against Salmonella infection, maintaining macrophage antimicrobial efficacy through the regulation of iron metabolism, whereas defects in NOS2 affect iron metabolism and FPN1 expression, thereby reducing host resistance to infection (83). In addition, overexpression of FPN can inhibit the proliferation of Mycobacterium tuberculosis in macrophages and simultaneously reduce the upregulation of inducible nitric oxide synthase (iNOS) protein expression and its bactericidal activity, and IFN-γ can also reverse a series of reactions triggered by overexpression of FPN (84). It has also been demonstrated that a lack of the hemochromatosis gene (HFE) deficiency leads to low iron in macrophages to inhibit inflammatory factor production, whereas high iron accumulation triggered by FPN downregulation acts as a pro-inflammatory signal to activate cytokine expression (85). Macrophages rely on NOX2-containing NADPH oxidase to generate large amounts of ROS to constitute an antimicrobial infection mechanism and achieve a balance between pathogen clearance and inflammatory damage regulation, precisely regulating the immune system (86). ROS produced by macrophages can effectively restore lysosomal function, improve autophagy, activate M1-type macrophages, and promote the production of inflammatory factors, which creates an opportunity and environment for ferroptosis (87).

Macrophages can be categorized into M1-type macrophages, which have pro-inflammatory properties and are involved in the clearance of pathogens but may cause tissue damage. M2-type macrophages have functions such as anti-inflammation and repair and are involved in fibrosis, wound healing, and immunosuppression (88, 89). Macrophage phenotypic polarization is regulated by specific cytokines. M1-type macrophages are driven by pro-inflammatory factors such as IFN-γ and TNF, activate STAT1, IRF5, and NF-κB pathways, and inhibit ferroptosis-induced lipid peroxidation through expression of inducible iNOS and production of NO, which in turn inhibits ferroptosis-induced lipid peroxidation, and their iron retention mechanism reduces the tissue iron concentration for bacteriostatic and antitumor activity (90, 91); M2 macrophages depend on anti-inflammatory factors such as IL-4, IL-13, IL-10, and other anti-inflammatory factors to activate and mediate STAT6, IRF4, and PPARγ signaling, whereas M2 macrophages release iron to promote microenvironmental cell growth and tissue repair while favoring tumor proliferation (92). M1 macrophages present a low expression of FPN, CD163, and HO-1, accompanied by high ferritin expression to maintain iron retention; the M2 type, in contrast, shows a high expression of all three with reduced ferritin, promoting iron metabolic export (93). Different studies have shown that differences in macrophage activation status significantly affect their iron metabolism status and ferroptosis sensitivity. RECALCATI et al. (94) showed that M2 macrophages exhibit more active iron metabolism and are more pro-proliferative compared with M1 macrophages. Agoro et al. (95) demonstrated that, in addition to promoting M2 polarization and inhibiting M1 pro-inflammatory responses, iron overload also promotes iron metabolism and inhibits the M1 pro-inflammatory response by reducing NF-κB nuclear translocation, pro-inflammatory factors, and iron-modulating hormone expression and restores FPN. M1-type macrophages are less sensitive to ferroptosis induced by the GPX4 inhibitor RSL3, whereas M2-type macrophages are more susceptible to such inducers due to low iNOS expression (91). Jiahui et al. (96) demonstrated that exosomal CagA induces macrophage ferroptosis through dysregulation of iron homeostasis and modulation of ferroptosis-associated genes, consequently disrupting GSH metabolism and ROS equilibrium. Furthermore, it activates the JAK-STAT pathway, driving M1 polarization with upregulation of iNOS and IL-1β. In iron-loaded M2 macrophages, LXRα induces the expression of hepcidin and FPN through an iron-loading mechanism. It also plays a key role in modulating macrophage inflammatory activity and iron-mediated proinflammatory responses (97, 98). Interestingly, ferroptosis tumor cells can contribute to the polarization of M2-type macrophages to M1-type macrophages and modulate the antitumor effects of TAMs with tumor cells during radiotherapy and immunotherapy (99). Ferroptosis inhibitors were shown to alleviate macrophage senescence and inflammatory factor levels by inhibiting the expression of substances such as related proteins and genes and promoting GPX4 expression, revealing a potential mechanism of ferroptosis signaling antagonism (100).

### 3.2 The mechanism of ferroptosis in macrophages

**Figure 2:**
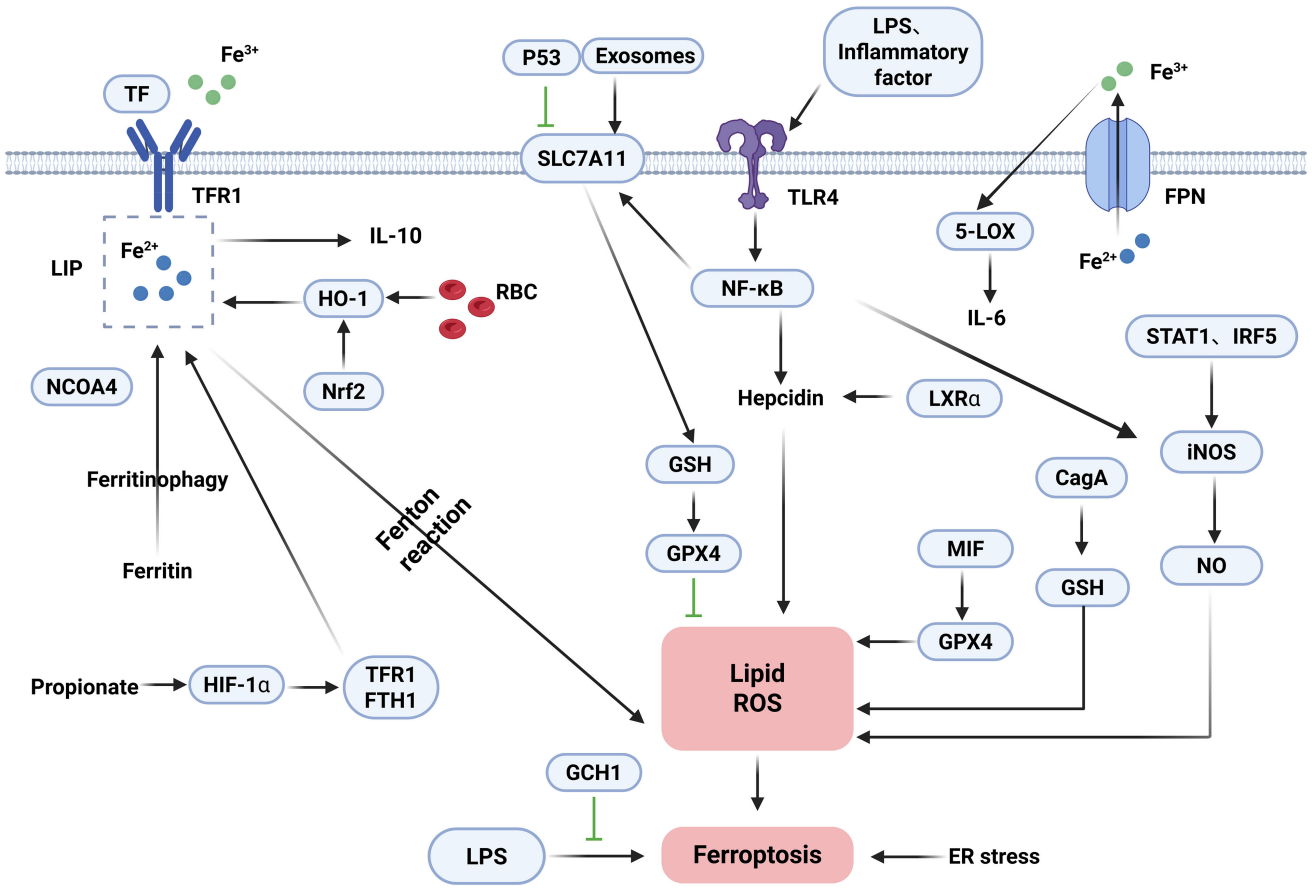
Mechanisms of ferroptosis in macrophages.

Iron can influence the development, function, and polarization of macrophages, which are critical for systemic iron homeostasis. There are two usual sources of iron in macrophages. First, macrophages produce iron by phagocytosis of damaged and decaying red blood cells (RBCs) and subsequent metabolic breakdown of intracellular heme by HO-1 (101). Secondly, extracellular iron binds to TF and enters macrophages via TFR1. Macrophages regulate iron distribution through the expression of FPN and ferritin, which are able to excrete excess iron out of the cell in a timely manner or store it in the Fe^3+^ form. Hepcidin regulates the flow of iron into the bloodstream by accelerating the degradation process of the iron export factor, FPN, in target cells. At the overall level, the maintenance of iron homeostasis relies on the regulatory axis formed by hepcidin and FPN (102). Specifically, hepcidin expression rises during iron overload, whereas iron export processes mediated by FPN are inhibited, so FPN expression is critical for regulating iron release from macrophages (103, 104). In addition, hepcidin autocrine secretion creates a vicious cycle of iron retention through the TLR4/NF-κB pathway, which is reinforced by ox-LDL and time-dose-dependent upregulation of hepcidin expression, ultimately leading to intracellular lipid deposition in macrophages (105). In addition to the hepcidin-protein regulatory axis, several molecular substances can influence iron content and iron homeostasis. NEDD4L exerts ferroptosis resistance by blocking iron-dependent oxidative damage by mediating the degradation of proteins with pro-iron deposition effects (106). HO-1 and BACH1 increase the amount of free iron in two different ways (33, 60). Under normal physiological conditions, iron levels in macrophages are regulated by a variety of regulatory factors to maintain a homeostatic state. However, under pathological conditions, if the iron load of macrophages exceeds their processing capacity, free radicals generated in the Fenton reaction may trigger iron deposition and lipid peroxidation phenomena, which may ultimately lead to cell death triggered by iron overload. Guo et al. (107) demonstrated that hepcidin was able to inhibit the iron export process of macrophages mediated by FPN, which then contributed to the intracellular iron level rise and drove osteoclast precursors to undergo proliferation and differentiation. In addition, iron overload regulates macrophage 5-lipoxygenase (5-LOX) bioactivity by enhancing macrophage adhesion to the nuclear membrane and generates inflammation by overexpression of inflammatory factors such as IL-6 in macrophages (108). Dmitry et al. (109) demonstrated that Nrf2, BACH1, and FPN are key regulators enhancing macrophage resistance to ferroptosis. Inhibition of BACH1 reduces unstable LIP levels and lipid peroxidation. Conversely, suppression of FPN or Nrf2 increases LIP and lipid peroxidation, sensitizing cells to ferroptosis upon RSL3 treatment. Iron also affects macrophage function and regulates macrophage polarization. Macrophages show a dramatic increase in ferrous iron and lipid peroxidation in the early stages of infection, which returns to normal in the late stages, and the addition of ferroptosis inducers is effective in inhibiting bacteria (110). The antioxidant function of GPX4 is essential for suppressing ferroptosis in macrophages. Paradoxically, macrophages confer tolerance to lipid peroxidation-driven ferroptosis in the absence of GPX4 (111). Xia et al. (112) demonstrated that iron is conducted through cellular signaling pathways such as MAPK, NF-κB, and ATF4, affects cellular metabolic processes such as glucose and lipids, and influences macrophage polarization through epigenetic regulatory methods such as miRNA regulation, DNA, and histone modification. GCH1 suppresses LPS-induced macrophage ferroptosis by activating the AMPK pathway, while concurrently reducing polarization and pro-inflammatory cytokine levels (113). Propionate, a therapeutic short-chain fatty acid for IBD, modulates iron homeostasis and inhibits ferroptosis through regulating TFR1/FTH1 expression. This promotes M2 macrophage polarization, thereby decreasing pro-inflammatory factor secretion and enhancing epithelial regeneration (114). RECALCATI indicated that (94) ferritin (FT) was highly expressed in M1-type macrophages, whereas the expression levels of FPN, HO-1, and TFR1 were relatively low. In contrast, FT expression was low in M2-type macrophages, whereas FPN, HO-1, and TFR1 showed a high expression. Studies indicate that exosomes from adipose tissue macrophages (ATMs) induce ferroptosis by targeting SLC7A11 to inhibit GSH synthesis (115). The ER and mitochondria serve as primary sites for ROS generation and metabolism. Silica triggers ferroptosis in murine macrophages and amplifies inflammatory responses, whereas Wnt/Ca^2+^ signaling activation exacerbates ER stress and mitochondrial redox imbalance by downregulating GPX4 and SLC7A11 expression (116). Notably, LPS or inflammatory cytokines upregulate SLC7A11 expression by activating the TLR4/NF-κB pathway. This promotes GSH synthesis and enhances GPX4 activity, enabling the clearance of lipid peroxides and supporting the survival of M1 macrophages. In contrast, M2 macrophages lack sustained NF-κB activation, resulting in lower SLC7A11 expression. Consequently, M2 cells primarily rely on GPX4 to directly reduce lipid peroxides. When GPX4 activity is inhibited, M2 macrophages become significantly more susceptible to ferroptosis than M1 macrophages (117, 118). Pannexin 1, a channel protein mediating transmembrane transport, is a key therapeutic target involved in apoptosis and the regulation of IBD. Pannexin 1 deficiency promotes M2-like polarization while inhibiting M1-like polarization. It also increases GPX4 expression in M2-like macrophages, establishing an anti-inflammatory and antioxidant positive feedback loop (119). Inhibiting P53 reduces ROS and lipid peroxidation. It also restores the levels of SLC7A11, GPX4, and GSH. Brucella infection triggers ferroptosis in macrophages. This process is regulated through the P53-SLC7A11-GPX4/GSH pathway and suppresses intracellular bacterial survival (120). In conclusion, ferroptosis and polarization in macrophages are caused by multiple mechanisms such as iron metabolism, GSH depletion, and GPX4 inactivation. Moreover, in different stages of macrophage polarization, the expression pattern of iron-related genes will be adjusted accordingly. The detailed mechanisms of ferroptosis in macrophages are shown in **Figure 2**.

## 4 Effect of macrophage ferroptosis in IBD and inflammation-cancer transformation

### 4.1 Macrophage ferroptosis promotes IBD progression

Macrophages participate in intrinsic immune defense through phagocytosis and secretion of inflammatory mediators, and iron homeostasis is of dual significance in maintaining macrophage physiological function and IBD progression. Iron overload in the gastrointestinal tract triggers the Fenton reaction and Haber–Weiss reaction, leading to excessive accumulation of ROS, which destroys membrane structure by oxidizing unsaturated fatty acids in the cell membrane and triggers mitochondria-endoplasmic reticulum dysfunction, which induces the expression of apoptotic proteins, such as caspases, and necrotic proteins, resulting in death and inflammatory deterioration of IECs damage. At the same time, an imbalance of iron metabolism enhances the ability of pathogenic microorganisms to adhere and invade, destroys the balance of intestinal flora, and triggers clinical symptoms such as abdominal pain and diarrhea (121). Exogenous iron can regulate 5-LOX activity and induce IL-6 expression by enhancing macrophage nuclear membrane binding capacity, thus confirming the functional regulation of iron overload on macrophages. Thus, it has been shown that iron specifically accumulates within inflammation-activated colonic macrophages in a UC mouse model (122). In addition, the mechanism of upregulation of colonic FPN expression in UC may involve hepcidin-mediated regulation of iron metabolism. As a major regulatory hormone produced by the liver in response to high iron load and inflammatory stimulation, hepcidin induces FPN1 to inhibit intestinal iron uptake and reduces iron release from macrophages during iron overload states (123, 124). In humoral immunity, IECs secrete ferritin by extracellular vesicles (EV). Excessive ferritin uptake increases cellular metabolic load, leading to iron accumulation in macrophages. The resulting iron overload condition promotes oxidative stress and inflammatory responses in these cells, thereby exacerbating intestinal inflammation (125). In addition to iron overload, iron can lead to ROS accumulation via the Fenton reaction, which causes lipid peroxidation and redox imbalance to induce macrophage ferroptosis, leading to intestinal epithelial injury. KAPRALOV et al. (91) revealed a novel redox mechanism for the regulation of ferroptosis under pathological conditions, whereby M1-type macrophages are highly susceptible to ferroptosis and thus produce inflammatory mediators, leading to the development of IBD. It was also found that given that inflammation-activated macrophages all showed a specific elevation of ferrous iron and that changes in intracellular ferrous iron levels may affect multiple metabolic processes, which in turn alter macrophage polarization status. Iron overload induces ROS generation, and ROS further mediates a significant upregulation of p53 protein expression and an enhancement of its acetylation modification level. This molecular regulation directly triggers the polarization of M1-type macrophages toward pro-inflammatory directions and accelerates the progression of IBD (126). It was shown that the STAT1 signaling pathway also regulates M1 macrophage polarization, whereas iron is mediated by blocking IFNγ-induced STAT1 phosphorylation, which in turn reduces iNOS expression and M1-associated cytokine levels (127). Another study demonstrated that a new substance, mineralized liposome CLF, could reduce iron accumulation in intestinal cells and effectively inhibit the ferroptosis process by re-establishing the GSH/GPX antioxidant system and decreasing macrophage ROS production and lipid peroxidation levels. At the same time, it reduces the susceptibility of M2-type macrophages to ferroptosis and protects polarized macrophages from ferroptosis depletion (17). γ-Glutamylcysteine (γGC) plays a central role in antioxidant and anti-inflammatory processes by regulating intracellular GSH levels. During IBD progression, γGC deficiency induces GSH depletion, consequently triggering macrophage ferroptosis and M1-type polarization, which collectively exacerbate intestinal inflammation. The engineered γGC-loaded microparticle (γGC-MP) delivery system suppresses ferroptosis through dual modulation: downregulating TNF-α and upregulating cytoprotective proteins. This coordinated action modulates the PI3K/AKT signaling pathway, ultimately promoting intestinal barrier restoration and ameliorating inflammation (128). Magnolin has a significant efficacy in alleviating DSS-induced IBD. It regulates macrophage polarization by inhibiting ALOX5, thereby reducing inflammatory cytokines and suppressing ferroptosis in IECs (129). To sum up, these results suggest a potential regulatory mechanism for macrophage ferroptosis in IBD.

### 4.2 Macrophage ferroptosis promotes the progression of inflammation-cancer transformation

**Figure 3:**
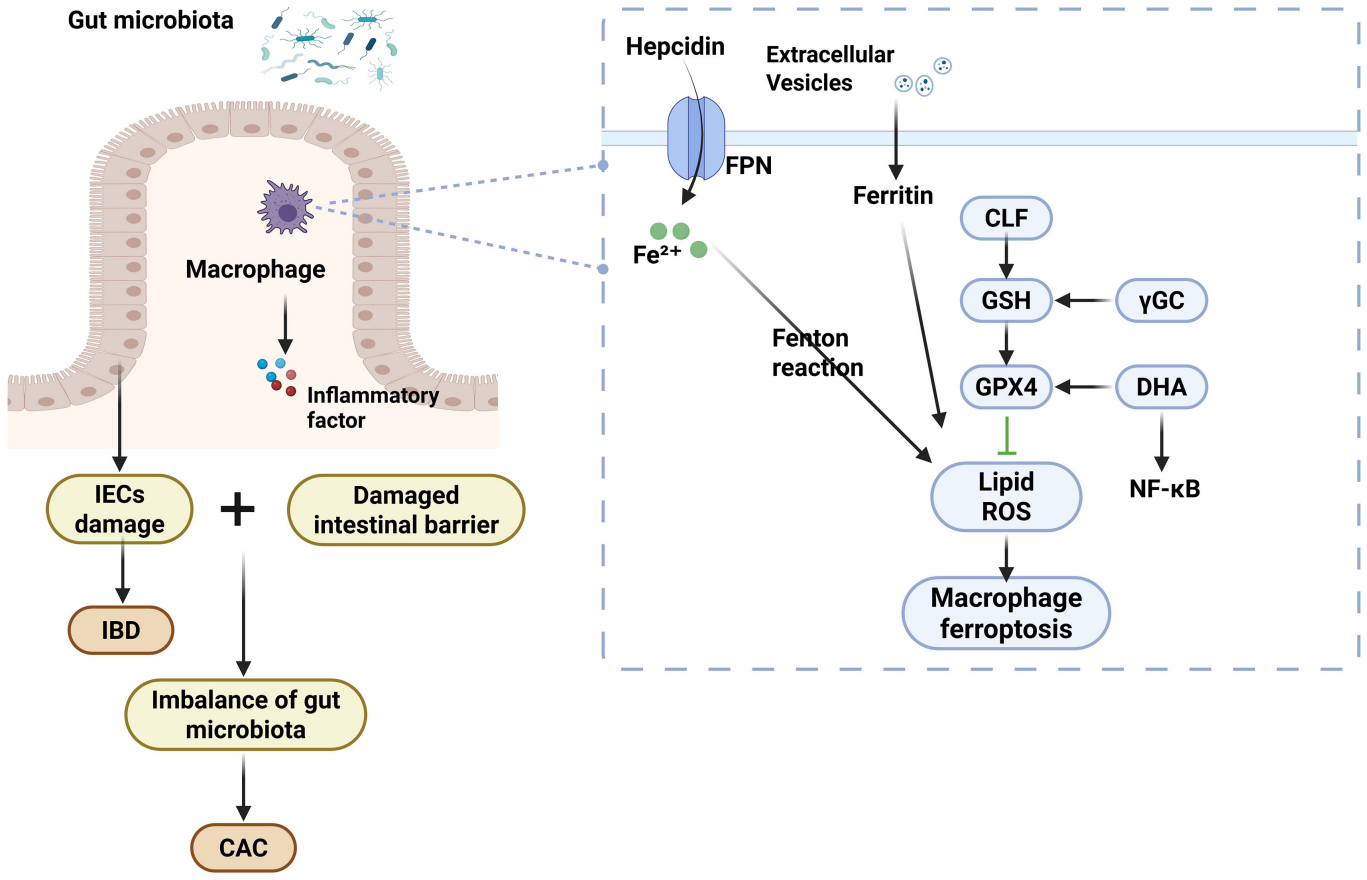
The mechanisms of macrophages’ ferroptosis are shown in IBD and inflammation-cancer transformation.

Epidemiological and related studies have shown that colorectal cancer risk is significantly elevated in IBD patients with long-term colonic involvement due to the recurrent nature of IBD and the repeated damage and repair of the intestinal mucosa. Patients with IBD-associated colorectal cancers are diagnosed at an advanced stage and have a poor prognosis (2, 130). Animal experiments have confirmed that inflammatory cell infiltration exacerbates the malignant progression of colitis-associated colorectal cancer. Therefore, balancing the bidirectional role of ferroptosis between tumor cells and immune cells is crucial, and there is an urgent need to develop more effective early risk assessment and therapeutic targets to improve the status quo. Chronic recurrent inflammation, through a pathological process triggered by sustained induction of IEC DNA damage, induces cellular alterations and immune responses and promotes tissue repair and cell proliferation, leading to colitis-associated colon cancer (CAC). The development of CAC is associated with a variety of *in vivo* microenvironments, including cellular metabolism, immune cells, and microbes (131). Macrophage infiltration and its polarization create a microenvironment in the transition from inflammation to tumor by exacerbating inflammation-associated mucosal injury and can regulate tumorigenesis through aberrant activation of multiple inflammatory pathways and dysregulated secretion of proinflammatory factors (132–134). M1-type macrophage polarization exacerbates inflammatory responses and induces gene mutations, whereas M2-type macrophage polarization partially antagonizes the pro-inflammatory effects of M1-type, but the overall polarization imbalance still promotes malignant transformation of epithelial cells. This pleiotropic mechanism of action makes macrophages an important regulatory node linking chronic inflammation to tumorigenesis (134–136). Tumor-associated macrophages (TAMs), as a type of highly plastic immune cell, have the dual functions of promoting and suppressing cancer. Studies have shown that its recruitment process is closely related to the antitumor immune response in the context of chronic inflammation, and TAM plays an important role in promoting tumor cell invasion of parenchymal tissues, enhancing tumor cell endocytosis, and other key aspects of tumor progression by modulating the immune response cascade (137, 138). The structurally intact intestinal epithelium is a key foundation for resistance to tissue damage and inflammation. Iron-dependent lipid peroxidation-driven cell death can disrupt intestinal barrier function, and the ROS it generates accelerates intestinal mucosal injury and promotes IBD development. Activation of intestinal cell proliferation pathways and downregulation of ferroptosis-related gene expression effectively inhibit the ferroptosis cascade, thereby significantly alleviating the transition from colitis to CAC (139–142). As nanotechnology for tumor therapy advances, researchers have developed a novel combination strategy. By depleting GSH and accumulating ROS, this method causes ferroptosis. At the same time, it promotes macrophage polarization toward an antitumor phenotype, effectively remodeling the immunosuppressive tumor microenvironment (143). Studies have shown that dihydroartemisinin (DHA) promotes iron accumulation and suppresses GPX4, leading to ROS accumulation and NF-κB activation. This induces ferroptosis in macrophages. Additionally, DHA-treated TAMs exert antitumor effects by polarizing into a pro-inflammatory phenotype through NF-κB activation (144). In addition, many pieces of evidence suggest that ferroptosis-related biomarkers have potential applications in the clinical management of colon cancer (145). Gut flora-mediated cancer therapies may act through ferroptosis inhibition of effector immune cells or enhancement of ferroptosis processes in immunosuppressed cells. Inhibition of ferroptosis in regulatory macrophages may also impair their immunosuppressive function and thus inhibit tumor progression (142). Therefore, inhibition of macrophage ferroptosis is important not only for alleviating IBD but also for preventing the development of CAC. The mechanisms of macrophages’ ferroptosis are shown in IBD and inflammation-cancer transformation **Figure 3**.

## 5 Summary and outlook

In this paper, firstly, the concept of ferroptosis and its regulatory mechanism were elaborated in detail, and then the relationship between ferroptosis and macrophages with their polarization was discussed through the perspective of iron metabolism and finally led to the relationship between macrophage ferroptosis and IBD. Ferroptosis is a novel type of programmed cell death caused by lipid peroxidation, the pathogenesis of which is generally due to iron overload, GSH depletion, GPX4 inactivation, and lipid peroxidation, and it is regulated by a variety of cellular metabolic activities and signaling pathways. Macrophages, as a key component of innate immunity, have multiple important biological functions. Iron affects macrophage development, function, and polarization, and macrophages are critical for the regulation of systemic iron homeostasis. Macrophage ferroptosis has now been shown to be involved in pathogenesis during the progression of various diseases such as atherosclerosis, tumors, and sepsis in arteries. The intestinal flora modulates ferroptosis in immune cells by many mechanisms, but the specific pathways of action by which they inhibit or promote ferroptosis have not been clarified. There is a growing body of research suggesting that ferroptosis and macrophages play important roles in IBD, such as maintaining immune and inflammatory homeostasis. Therefore, macrophage ferroptosis is expected to be a new target for the treatment of IBD.

Currently, studies on ferroptosis in IBD are mainly focused on IECs, and the role of ferroptosis in other relevant immune cells in IBD deserves to be further explored, and the relationship between iron homeostasis, immune homeostasis, and the various symptoms of IBD needs to be deeply explored. Although many advances have been made in the study of macrophage ferroptosis, few studies have focused on the impact of macrophage ferroptosis and IBD. The exact mechanism of how macrophage ferroptosis affects IBD through IECs, gut flora, and other directions remains imperfect, and how macrophage and ferroptosis crosstalk with each other has not yet been articulated. In addition, macrophage ferroptosis has been studied more at the gene and molecular levels, but less at the cellular and animal levels, and how to treat IBD in the clinic through its specific mechanism, and translate it into practical results. Therefore, in future studies, we can further confirm the specific regulatory mechanism of macrophage ferroptosis in IBD and explore the signaling pathways and cellular metabolic activities in more detail, which will be important for revealing the evolution of the disease and developing the target macrophage ferroptosis. This is of great clinical significance for revealing the mechanism of disease evolution and developing novel drugs and therapeutics targeting macrophage ferroptosis.
